# Multiparametric cardiac magnetic resonance evaluation of myocardial involvement in Duchenne muscular dystrophy: A case report

**DOI:** 10.1016/j.radcr.2025.09.079

**Published:** 2025-10-24

**Authors:** Hiroto Takamure, Seitaro Oda, Masafumi Kidoh, Yukako Ichiguchi, Keiko Nomura, Shinsuke Hanatani, Kimitoshi Nakamura, Kenichi Tsujita, Toshinori Hirai

**Affiliations:** a Department of Diagnostic Radiology, Kumamoto University, 1-1-1 Honjo, Chuo-ku, Kumamoto, 860-8556, Japan; b Department of Pediatrics, and Kumamoto University, 1-1-1 Honjo, Chuo-ku, Kumamoto, 860-8556, Japan; c Department of Cardiovascular Medicine, Faculty of Life Sciences, Kumamoto University, 1-1-1 Honjo, Chuo-ku, Kumamoto, 860-8556, Japan

**Keywords:** Duchenne muscular dystrophy, Cardiac magnetic resonance imaging, T1 mapping

## Abstract

Duchenne muscular dystrophy (DMD) is a progressive X-linked disorder characterized by degeneration of both skeletal and cardiac muscles. Myocardial involvement is a leading cause of mortality and often progresses subclinically due to patients’ limited physical activity. We report the case of a 15-year-old boy with DMD who exhibited asymptomatic left ventricular dysfunction. Cardiac magnetic resonance imaging (CMR) revealed diffuse systolic impairment with reduced circumferential strain. Multiparametric CMR demonstrated elevated native T1 and extracellular volume (ECV), indicative of diffuse myocardial fibrosis, along with prolonged T2 values suggestive of myocardial edema. Subepicardial fibrosis was identified on late gadolinium enhancement (LGE) imaging. These findings demonstrate the utility of advanced CMR techniques, including quantitative myocardial mapping and myocardial strain analysis, for comprehensive evaluation of DMD-associated cardiomyopathy. Multiparametric CMR allows for detailed myocardial characterization and may facilitate early therapeutic intervention prior to the onset of overt heart failure, thereby improving clinical management in patients with DMD.

## Introduction

Duchenne muscular dystrophy (DMD) is a progressive X-linked neuromuscular disorder characterized by the absence of dystrophin, leading to degeneration of skeletal, respiratory, and cardiac muscles. Cardiac complications are a major cause of death in DMD, typically presenting as progressive subclinical dilated cardiomyopathy (DCM), referred to as DMD-associated cardiomyopathy. Myocardial involvement often occurs before clinical symptoms due to reduced physical activity; thus, regular cardiac assessment is essential regardless of symptomatology.

Late gadolinium enhancement (LGE) on cardiac magnetic resonance imaging (CMR) is useful for detecting myocardial fibrosis in DMD, although it may not be sensitive to early myocardial changes. Recent studies have reported the utility of T1 and T2 mapping and myocardial strain analysis for earlier detection of myocardial involvement. Here, we present a case in which multiparametric CMR provided valuable insights into myocardial tissue characterization in a patient with DMD.

## Case report

**Fig. 1:**
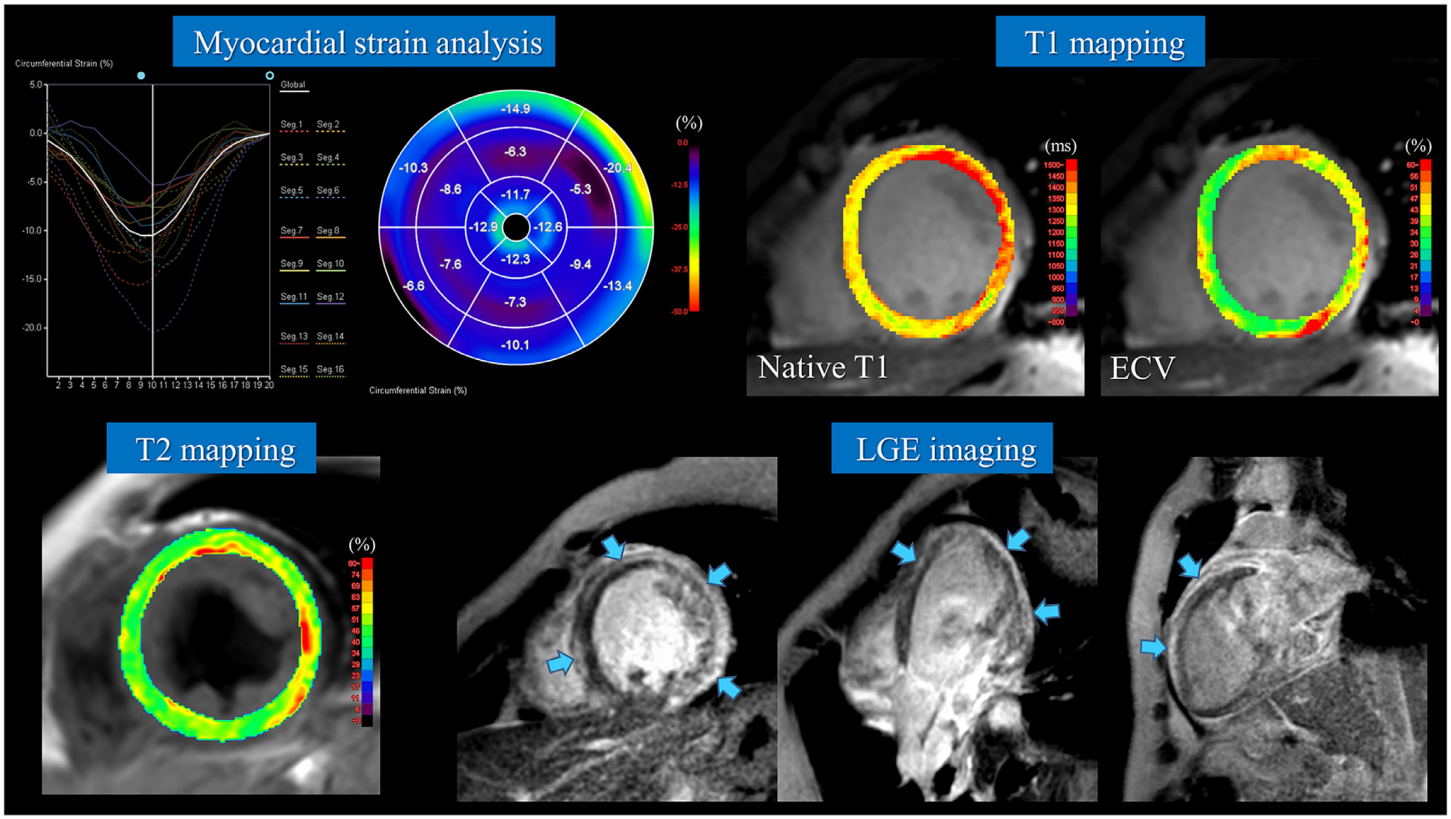
Multiparametric CMR of DMD-associated cardiomyopathy. Feature-tracking strain analysis showed reduced peak circumferential strain and marked temporal dispersion. T1 mapping demonstrated prolonged native T1 (1450 ms; reference: 1230-1250 ms) and elevated ECV (39%; reference: 23%-28%). T2 mapping revealed prolonged native T2 (53 ms; reference: 40-45 ms), suggestive of myocardial edema. LGE imaging showed a ring-like distribution of extensive subepicardial fibrosis in the LV myocardium. (MRI system: Ingenia CX, R5.4; Philips Healthcare, and image analysis workstation: Ziostation REVORAS; Ziosoft).

A 15-year-old boy with a diagnosis of Duchenne muscular dystrophy (DMD) had been followed since the age of 8 months. He showed progressive limb muscle weakness, lost ambulation at the age of 9, and became unable to turn in bed by age 11. He currently uses a power wheelchair for mobility. During routine follow-up, transthoracic echocardiography revealed newly developed left ventricular (LV) dysfunction, and blood tests showed elevated levels of serum brain natriuretic peptide (BNP, 35.9 pg/mL) and high-sensitivity troponin T (0.0487 ng/mL). Cardiac magnetic resonance imaging (CMR) was subsequently performed (Fig. 1).

Cine CMR demonstrated LV dilation and heterogeneous wall thinning, with diffusely impaired LV wall motion and a reduced LV ejection fraction of 30%. Feature-tracking strain analysis revealed a decreased peak circumferential strain and marked temporal dispersion of peak timing. T1 mapping showed significantly prolonged native T1 values (1450 ms; institutional normal range: 1230-1250 ms) and elevated extracellular volume fraction (ECV: 39%; normal range: 23%-28%). While no abnormalities were noted on fat-suppressed T2-weighted imaging, T2 mapping revealed prolonged native T2 values (53 ms; institutional normal range: 40-45 ms), suggestive of myocardial edema. LGE imaging demonstrated a ring-like pattern of extensive subepicardial fibrosis in the LV myocardium. Based on these findings, the patient was diagnosed with DMD-associated cardiomyopathy, and optimal pharmacologic therapy, including an angiotensin-converting enzyme (ACE) inhibitor, a β-blocker, and a diuretic, was initiated. Following treatment, serum BNP levels returned to within the normal range.

## Discussion

Myocardial involvement in DMD is progressive and is pathologically characterized by cardiomyocyte necrosis, atrophy, and fibrosis [1]. Due to restricted physical activity, patients with DMD often remain asymptomatic until the cardiac disease has significantly progressed. Although regular echocardiographic evaluation is recommended, it has limited sensitivity for detecting early myocardial lesions [2]. Therefore, cardiac monitoring using CMR is warranted.

LGE imaging on CMR can visualize myocardial fibrosis associated with DMD. In DMD-associated cardiomyopathy, LGE typically first appears in the subepicardial region of the inferolateral wall of the left ventricle and subsequently progresses in a circumferential pattern [3]. T1 mapping offers a means to detect diffuse myocardial fibrosis that may not be apparent on LGE imaging. Even in patients with preserved LVEF and negative LGE, elevated native T1 and ECV values have been observed, suggesting the presence of subclinical myocardial involvement [4]. Furthermore, increased ECV has been shown to correlate significantly with reduced LVEF [5]. These findings indicate that T1 mapping may serve as a useful biomarker for the early diagnosis and therapeutic monitoring of myocardial injury in DMD. Prolongation of T2 relaxation times in the thigh muscles of patients with DMD has been previously reported and shown to correlate with disease progression [6]. Furthermore, alterations in myocardial T2 values in DMD patients have been reported to become more pronounced with increasing age and decreasing LVEF [7]. Sudeep et al. proposed a comprehensive myocardial tissue characterization using CMR-based T1, T2, and ECV mapping to evaluate the pathophysiology of myocardial involvement in DMD in detail [8]. In a cohort of 49 DMD patients, myocardial compositional changes—such as fibrosis, edema, and fatty infiltration—were classified and analyzed. The most prevalent finding was myocardial fibrosis, while T2 prolongation indicative of edema was more frequently observed in older patients and those with reduced LVEF. In most cases with elevated T2 values, myocardial abnormalities persisted, with normalization being rare. Fatty infiltration and fibro-fatty changes were identified in a minority of cases, suggesting their association with advanced disease stages. This study highlights the utility of noninvasive multiparametric CMR mapping in delineating the progression of DMD-associated cardiomyopathy and provides critical insights for identifying potential targets for future therapeutic intervention. In patients with DMD, myocardial strain analysis using CMR feature tracking has demonstrated a significant reduction in global circumferential strain compared with healthy controls, with particularly pronounced differences observed in the anterolateral, inferolateral, and inferior segments. Furthermore, CMR feature tracking has been shown to clearly detect differences in strain values between LGE-positive and LGE-negative regions, which cannot be readily distinguished by 2D speckle tracking echocardiography [9].

## Conclusion

As illustrated in this case, the integration of advanced multiparametric CMR techniques—including T1, T2, and ECV mapping, along with feature tracking-based myocardial strain analysis—into conventional LGE imaging may enhance early detection of myocardial injury, support longitudinal disease monitoring, and inform therapeutic decision-making in DMD-associated cardiomyopathy.

## Ethical approval

This study does not require institutional review board approval.

## Patient consent

Written informed consent was obtained from the patient's parents for anonymized patient information to be published in this article.
